# Focal cryosurgical ablation of the prostate: a single institute’s perspective

**DOI:** 10.1186/1471-2490-13-2

**Published:** 2013-01-11

**Authors:** Zachary Hale, Makito Miyake, Diego Aguilar Palacios, Charles J Rosser

**Affiliations:** 1 Section of Urologic Oncology, MD Anderson Cancer Center Orlando, 1400 S. Orange Ave, Orlando, FL, 32806, USA

**Keywords:** Prostate cancer, Therapy, Focal, Cryoablation

## Abstract

**Background:**

With the stage migration of prostate cancer witnessed in the late 1990’s and early 2000’s along with the persistent morbidities associated with prostatectomy and radiation therapy, the concept of focal prostate cancer treatment remains quite attractive. Herein we evaluate the tolerability and non-oncologic outcomes of a highly select cohort of men that underwent focal cryoablation of the prostate for the treatment of localized prostate cancer.

**Methods:**

Pre-operatively, erectile function was assessed by SHIM questionnaire while voiding symptoms were assessed by AUA symptom score. Twenty-six highly select patients (23 low-risk prostate cancer and 3 intermediate-risk prostate cancer) with documented minimal disease on saturation prostate biopsy underwent focal cryoablation of the prostate (24 hemi-ablation and 2 subtotal ablation). Subsequently, serum PSAs were obtained every 3 months for 2 years and then every 6 months thereafter. PSA failure was defined as an increase of 0.50 ng/ml over nadir. Mean follow-up was 19.1 months. Subjective assessment of erectile function and voiding was assessed post-operatively at each visit.

**Results:**

Based on our PSA failure definition, 11.5% (3 patients) of the cohort experienced biochemical failure. In two of the three patients, localized disease was detected on subsequent transrectal ultrasound guided biopsy. These two patients went on to have favorable PSA nadirs after undergoing conventional definitive therapy (one patient had external beam radiation and one patient had whole gland cryoablation). Within the study cohort, 27% (7 patients) reported new post-operative erectile dysfunction requiring therapy while no patients reported new post-operative urinary incontinence or worsening of voiding symptoms.

**Conclusion:**

These preliminary results add to the expanding body of literature that the minimally invasive focal cryosurgical ablation of the prostate is a safe procedure with few side effects. The true extent of cancer control remains in question, but in highly select patients, favorable PSA kinetics have been demonstrated. If confirmed by further studies with long-term follow-up, this treatment approach could have a profound effect on prostate cancer management.

## Background

In the era of PSA screening for prostate cancer, we have witnessed a stage and grade migration leading to a high proportion of men diagnosed with small volume, low-risk prostate cancer (*i.e.,* serum PSA < 10, Gleason 6, T1c) [1,2]. Due to the non-aggressive nature of some small volume, low-risk prostate cancers, the ideal management option is not known. Recognized management options for this cohort include: active surveillance, brachytherapy, external beam radiation therapy, total gland cryoablation and prostatectomy (retropubic, perineal and robotic). In the highly select patient, cancer specific survival employing any of these treatment options is excellent, however morbidity from these interventions can be significant. Thus, the idea of treating only the cancer within the prostate and sparing the non-cancerous tissue is quite appealing, yet controversial.

As defined by the International Task Force on Prostate Cancer and the Focal Lesion Paradigm, the goal of focal therapy for prostate cancer would be to “selectively ablate(s) known disease and preserve(s) existing functions, with the overall objective of minimizing lifetime morbidity without compromising life expectancy” [3]. The Task Force reported that the ideal candidate for focal therapy is one with low-risk prostate cancer. These selection criteria are illustrated in Table 1[4]. Focal cryoablation of the prostate, first reported by Onik *et al.*, is a minimally invasive treatment modality with encouraging preliminary biochemical efficacy results and reduced morbidity compared to conventional treatment options [5]. Based on the above findings, we have selectively performed focal cryoablation of the prostate in 26 patients over the past six years. Herein we evaluate the tolerability and non-oncologic outcomes of a highly select cohort of then who underwent focal cryoablation of the prostate for the treatment of localized prostate cancer. 

**Table 1:** Ideal candidate for focal therapy

	
Serum PSA	PSA < 10 ng/mL, PSAD < 0.15 ng/mL/g
Clinical stage	T1NxMx or T2aNxMx
Pathologic evaluation/Gleason score**	3 + 3 or less (no grade 4 or 5)
	No more than 2 adjacent regions positive for cancer
	Total length of cancer <10 mm total and <7 mm in any 1 core; <1/3 of cores positive for cancer

* Modified from Sartor *et al.*, 2008 [4] **10 core minimum biopsy schema, plus 2 additional cores for every 10 g of prostate >40 g (max 18 cores).

## Methods

After MD Anderson Cancer Center Orlando Institutional Review Board approval, medical records of 26 men who had undergone focal cryoablation from January 2006 to March 2012 were extensively reviewed. Preferentially, low-risk prostate cancer patients (*i.e.*, serum PSA </= 10.0 ng/ml, Gleason <7, < cT2b; n = 23), were considered for focal ablation, while only three intermediate-risk prostate cancer patients (*i.e.,* serum PSA 10-20 ng/ml; n = 2 or Gleason score 7; n = 1) underwent focal cryoablation of the prostate. Clinic and hospital records were reviewed for patient demographics, disease characteristics, pre-operative American Urological Association (AUA) symptom index questionnaire and Sexual Health Inventory for Men (SHIM), post-operative voiding and erectile function and follow-up (Table 2). 

**Table 2:** Demographics and preoperative characteristics

** Variables**	
**Age, years, median (range)**	65 (55-74)
**Race**	**n (%)**
White	23 (88)
Hispanic	1 (4)
Black	2 (8)
**Pretreatment clinical stage**	**n (%)**
T1c	26 (100)
**Gleason score in entry biopsy**	**n (%)**
3 + 3	25 (96)
3 + 4	1 (4)
**PSA at pre-operative evaluation**	**n (%)**
</= 10	24 (92)
10-20	2 (8)
**Entry staging biopsy (all patients)**	
Median performed, n (range)	40 (30-60)
Median cores positive for cancer, n (range)	4 (1-6)
Unilateral Cancer, n (%)	24 (92)
Bilateral Cancer, n (%)	2 (8)
**Preoperative urinary continence, n (%)**	26(100)
**Preoperative SHIM score median, n (range)**	20 (16-25)
**Mean follow-up, months**	19.1

Initially, 33 patients interested in focal cryoablation were seen in our outpatient clinic for evaluation of low-grade, low-stage prostate cancer confirmed on histologic examination of outside pathologic slides. On average, four weeks prior to focal cryoablation in the outpatient setting, all patients underwent a modification of a 3D mapping ultrasound guided transperineal prostate biopsy [6] under monitored anesthesia care (MAC) to confirm extent and location of tumor(s). Each biopsy specimen was labeled with location and orientation, which allowed precise localization of tumor burden within the prostate. The saturation prostate biopsies confirmed low volume disease in 26 patients who comprised our study cohort.

**Figure 1:**
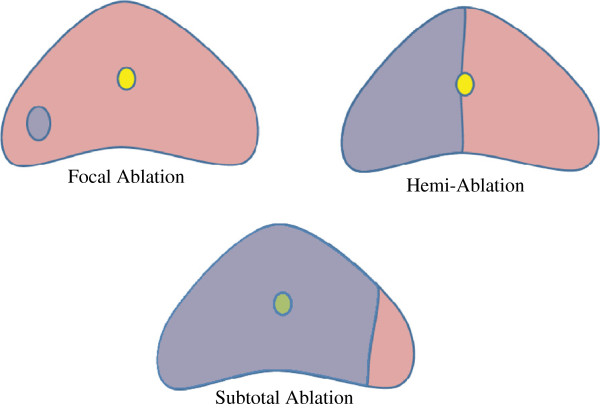
**Schematic depicting ‘focal’cryoablation. **Ablation can be of single target lesion, of specific side of prostate harboring the cancer or of nearly the entire gland but sparing the posterior lateral aspects were caversonal resides.

The Endocare’s Cryocare CS system with the variable probes along with the urethral warmer was utilized (median number of 3 probes) on all cases. Focal cryoablation was performed as previously described [5,7] by one surgeon (CJR) in an outpatient setting. Intra-operative flexible cystoscopy was reserved only for patients, who were noted to have severe voiding symptoms on their pre-operative evaluation or if correct probe placement could not be confirmed on real-time ultrasound. Injection of 30 ml of local anesthetic into Denoviller’s fascia to anesthetize and hydrodissect the prostate from the rectum prior to the initiation of the freeze cycles was employed in all cases. Twenty-four patients underwent hemi-ablative cryosurgery while the two with bilateral disease underwent subtotal cryosurgery with an attempt to spare the prostatic tissue that resides next to the cavernosal nerve (Figure 1). After the procedure, 25 patients were discharged from post anesthesia care unit to home with a Foley catheter in place for 7-10 days at which time all catheters were successfully removed in clinic. One patient was admitted for twenty-three hours observation due to social reasons. Four weeks after all procedures, the patients returned to clinic with a serum PSA. 

Serum PSAs were monitored every 3 months for 2 years then every 6 months thereafter. In addition, yearly digital rectal examinations (DRE) were performed. Biochemical failure (BCF) was defined as an increase in serum PSA of 0.5 ng/ml over nadir [8]. Patients with BCF and/or abnormal DRE were encouraged to undergo a repeat transrectal ultrasound needle guided biopsy of the prostate in hopes of identifying recurrent disease. Patients were considered to be potent if they were able to achieve erections sufficient for penetration and impotent if they were unable to achieve erections sufficient for penetration. Patients requiring any potency aids, including phosphodiesterase inhibitors, pumps, and vacuum devices, were noted. Patients were considered incontinent if they used any pads at any time. Mean follow-up time was 19.1 months (range 2-52 months). Follow-up data ≥1 year was available in 18 patients (69%), 1-2 years in 8 patients (31%), 2-5 years in 9 patients (35%).

## Results

Median prostatic volume was 33 grams. No complications were noted after 3D mapping ultrasound guided transperineal prostate biopsy. A mean of 35 cores were obtained in each patient during the saturation biopsy with a median of 4 cores per patient being positive for cancer. Only two patients were noted to have bilateral disease, with the majority of disease unilateral and no more than 2 cores positive on the contralateral side. The 24 (92%) patients, who had unilateral disease, underwent hemi-cryoablation of the prostate, whereas 2 (8%) patients had bilateral disease and underwent sub-total cryoablation of the prostate. Demographics and disease characteristics are summarized in Table 2. The median age of the patients was 65 years. Ninety-two percent of the patients were Caucasian. All patients had non-palpable T1c cancer and 25 (96%) had Gleason 3 + 3 (6) disease.

**Figure 2:**
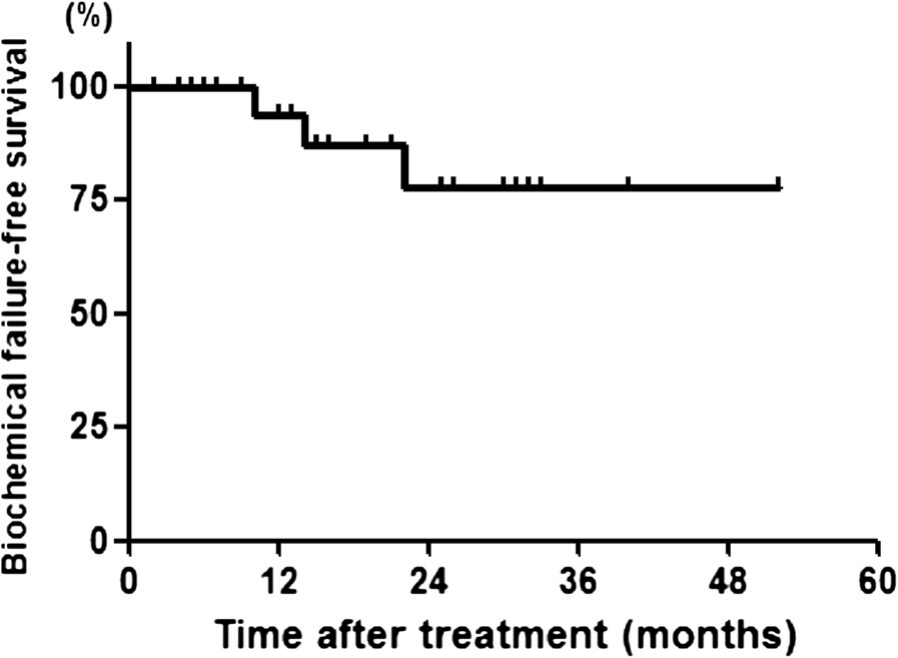
Biochemical disease free-survival of 26 patients treated with focal cryoablation of the prostate.

Three patients developed PSA recurrence in follow-up (Figure 2). Specifics related to the three patients (11.5%) with PSA recurrence are shown in Table 3. Only two of these patients (8%) had biopsy confirmed localized disease (Gleason 6). These two patients with documented local recurrence underwent salvage procedures (1-external beam radiation, 1-whole gland cryoablation). After salvage therapy, the serum PSA of these two patients decreased to favorable nadirs. Oncologic and functional outcomes are depicted in Table 4. There were no deaths, nor development of locally advanced/metastatic disease. 

**Table 3:** Patients with biochemical failure after focal cryoablation of the prostate

**Patient**	**Pre-op Gleason score**	**Pre-op PSA**	**Extent of ablation**	**PSA Nadir**	**Max PSA at failure**	**Biopsy proven**	**Subsequent treatment**	**Final PSA**	**Follow-up months**
		**(ng/ml)**		**(ng/ml)**	**(ng/ml)**	**Recurrence**		**(ng/ml)**	
1	6	8.8	Hemi-ablation	2.8	3.7	No	Watchful Waiting	3.3	31
2	6	5.3	Hemi-ablation	3.1	4.2	Yes	External beam radiation	0.4	18
3	6	4.5	Hemi-ablation	1.7	3.1	Yes	Whole gland cryoablation	0.2	15

**Table 4:** Oncologic and functional outcomes

**Variable**	**n (%)**
*Oncologic*	
Patients with serum PSA failure	3 (12)
Deaths	0 (0)
Metastasis	0 (0)
*Functional*	
Erectile Dysfunction Requiring Treatment	7 (27)
Impotent	0 (0)
Urinary Continence	26 (z100)
*Complications*	
Fistula	0 (0)
Urethral slough	0 (0)
Urinary Tract Infection (UTI)	1 (4)
Urinary retention	1 (4)
Rash	1(4)

The mean pre-operative SHIM score was 20. All patients underwent ‘nerve-sparing’ focal ablation. Post-operative erectile dysfunction, requiring treatment of any kind, occurred in 7 patients (27%). These patients had a significantly lower pre-operative SHIM score than the 19 patients who did not require any treatment for erectile dysfunction, 18.6 *vs.* 20.9, respectively, *p* = 0.015. No patient was found to be impotent. Seventeen patients had moderate voiding symptoms evident on AUA symptom score prior to surgery. No patient had severe voiding symptoms or incontinence prior to focal ablation. Urinary continence was maintained in all 26 patients. One of the patients requiring a subtotal cryoablation for bilateral disease developed postoperative urinary retention from a persistent median lobe after ablation (pre-operative AUA symptoms score 3). The median lobe required surgical resection as an outpatient to alleviate urinary retention. Focal cryoablation was well tolerated with only one patient developing a post-operative urinary tract infection (catheter associated) and another patient developing a transient rash possibly from pre-operative antibiotics.

## Discussion

Today, more low-volume, less aggressive prostatic cancers are being detected [1,2]. For example, in a retrospective study of 1,386 radical prostatectomies specimens, unilateral disease was present in approximately 20% of the specimens with many of these tumors being low volume [9]. These low-volume, low-risk prostate cancers, may not affect long-term survival, thus these men then have a reasonable choice to pursue active surveillance [10] or definitive treatment. While active surveillance may be associated with increased anxiety, definitive therapy may be associated with significant treatment related side effects (*e.g.,* voiding issues and erectile dysfunction) or the unnecessary treatment of a non-aggressive, non-lethal disease. The rationale for focal therapy is that it offers similar oncologic efficacy to definitive therapy but with reduced treatment morbidity, when applied to highly select patients [3,11].

In the current study, only highly select patients, identified by transperineal saturation prostate biopsy were offered focal cryoablation of the prostate. Intra-operative and immediate post-operative complications were minimal. Median PSA nadir of the cohort was 1.8ng/ml. Using a stringent PSA failure definition [8], three patients were noted to have biochemical failure with local failure documented in two out of the three patients on prostate biopsy. The patient without documented local failure but PSA failure was noted to have a subsequent reduction and stabilization in serum PSA. Of the two patients with the local failures, one was treated with whole gland cryoablation and one was treated with salvage external beam radiation therapy.

The median pre-operative AUA symptoms score of the cohort was nine. All patients were continent post-operatively. Except for the patient who developed post-operative urinary retention from an obstructing median lobe of the prostate, no one reported worsening voiding symptoms after focal cryoablation. All voiding symptoms resolved in the patient with urinary retention after he underwent a limited transurethral resection of the median lobe of the prostate. As for erectile function, the median pre-operative SHIM score was 20. Post-operative erectile dysfunction, requiring treatment of any kind, occurred in 7 patients (27%). These patients had a significantly lower pre-operative SHIM score than the 19 patients (73%) who did not require any treatment for erectile dysfunction. Thus a reduction in the erectile function may be expected in men undergoing focal ablation, who pre-operatively report reduced erections.

Numerous studies have reported their small experience in focal cryoablation of the prostate (Table 5) [7,12-15]. Our biochemical disease free survival rates, potency rates and incontinence rates are comparable to what has been reported in the literature. In a retrospective study of 73 low-intermediate risk patients (*i.e.,* PSA ≤ 20, Gleason score ≤ 7, clinical stage T1-T2b) whom underwent focal cryotherapy, after a median follow-up of 3.7 years, 12 patients (17%) had positive cancer biopsies postoperatively, but only one (1.4%) of those was in the ipsilateral, treated lobe. Biochemical disease free survival was not addressed in this study. All patients were continent after surgery, and of the patients who were potent preoperatively, 74% maintained their potency one year after the procedure, and that number grew with time [2]. One of the largest studies to address focal cryoablation of the prostate is from the national Cryo On-line Database (COLD) registry, an online retrospective database established to collect cryoablation outcomes from patients treated with primary, salvage and focal cryoablation. In this study, Ward *et al.* reported on 1160 focal cryoablation patients (median follow-up 1.8 years) in the database. Biochemical disease free survival was found to be 75.7%. Not all patients were biopsied postoperative, but those who were had a 3.7% rate of local failure. Urinary continence was high, at 98.4%, and spontaneous erections were maintained in 58.1% of patients [15]. 

**Table 5:** Focal cryosurgery outcomes

**Authors, dates**	**No. of pts**	**Follow-up years**	**Biochemical failure definition**	**Biochemical disease -free survival**	**Biopsy proven ipsilateral disease**	**Potency**	**Continence**	**Voiding symptoms**
Lambert *et al.*, 2007 [11]	25	2.3 (median)	ASTRO criteria	84% (PSA Nadir >50%)	4%	71%	100%	N/A
Ellis *et al.*, 2007 [12]	60	1.3 (mean)	ASTRO criteria	80% (ASTRO)	1.7%	70.6% @ 1yr	96.4%	N/A
Onik *et al.*, 2008 [7]	48	4.5 (mean)	ASTRO criteria	94% (ASTRO)	0%	90%	100%	N/A
Truesdale *et al.,* 2010 [13]	77	2 (median)	Phoenix criteria	73%	4%	N/A	N/A	N/A
Ward *et al.*, 2012 [14]	1160	1.8 (mean)	ASTRO criteria	75.7% (ASTRO) @ 3yr	3.7% (Unsure Laterality)	58.1%	98.4%	1.1%^^^
Bahn *et al.*, 2012 [2]	73	3.7 (median)	ASTRO criteria	N/A	1%	86%	100%	N/A
Present Study	26	1.6 (mean)	0.5 ng/ml over nadir [8]	88%	0%	73%*	100%	4%^^^

ASTRO criteria, 3 consecutive rises in the serum PSA after the post-radiation PSA nadir; Phoenix criteria, serum PSA nadir post-radiation + 2 ng/ml.

*, Remainder 27% potent with the use of pharmacologic or mechanical aids.

^, urinary retention.

Though the idea of only treating the cancer within the prostate and sparing the non-cancerous tissue is quite appealing, the medical community must continue to push to gather higher level of evidence on key issues related to focal therapy prior to widely offering this novel technology. These key issues include: 1) can we accurately identify index lesions by extensive mapping of the prostate, 2) can we reliably image cancers within the prostate, 3) what is the long-term efficacy of the technology to eradicate cancer and 4) how to follow-up of patients treated with focal therapy [11]. Hopefully, ongoing trials related to saturation prostate biopsy, prostate imaging, and focal therapy will provide the much needed evidence that focal therapy of the prostate is an oncologically effective therapy for highly-select patients interested in preserving urinary and erectile function.

Our study has several limitations. First, it is a small retrospective study from a single surgeon. Second, follow-up, though longer than in most focal cryoablation studies, was limited (mean follow-up of 19.1 months). Next, pre-operative and post-operative imaging was not incorporated into our algorithm due to the lack of accuracy of current imaging modalities to identify focal lesions within the prostate. Lastly, serum PSAs and digital rectal examinations were the only methods employed to monitor treatment efficacy. Though PSA and digital rectal examination are excellent means to follow-up patients after prostatectomy, whole gland irradiation and whole gland cryoablation, PSA and digital rectal examination after focal therapy may underestimate the extent of persistent diseases. PSA and digital rectal examination were shown to be inaccurate in following low-risk prostate cancer patients on a surveillance protocol, thus most surveillance protocols will incorporate transrectal ultrasound needle guided prostate biopsies in follow-up [16]. Moving forward, attention must be given to offering prostate biopsies to patients treated with focal therapy.

## Conclusions

In conclusion, these preliminary results add to the expanding body of literature that the minimally invasive focal cryosurgical ablation is a safe procedure with few side effects. The true extent of cancer control remains in question, but in highly select patients, favorable PSA kinetics have been demonstrated. Focal cryotherapy may fill a void in the management of low-risk prostate cancer patients, who refuse active surveillance and who want to avoid other definitive therapeutic options (prostatectomy, external beam radiation, brachytherapy and whole gland cryoablation) with potential increased risks of morbidity.

## Competing interests

The authors declare that they have no competing interests.

## Authors’ contributions

ZH, BS - Acquisition of data, statistical analysis and drafting manuscript. MM, MD, PhD - Statistical analysis and drafting of manuscript. DAP, MD - Acquisition of data and drafting manuscript. CJR, MD, MBA - Study concept and design, drafting of manuscript. All authors have read and approved the final manuscript.

## Pre-publication history

The pre-publication history for this paper can be accessed here:

http://www.biomedcentral.com/1471-2490/13/2/prepub
